# *N*-(3-Chloro­phen­yl)-3-nitro­pyridin-2-amine

**DOI:** 10.1107/S1600536811044011

**Published:** 2011-10-29

**Authors:** Aina Mardia Akhmad Aznan, Zanariah Abdullah, Seik Weng Ng, Edward R. T. Tiekink

**Affiliations:** a Department of Chemistry, University of Malaya, 50603 Kuala Lumpur, Malaysia; b Chemistry Department, Faculty of Science, King Abdulaziz University, PO Box 80203 Jeddah, Saudi Arabia

## Abstract

The dihedral angle between the benzene and pyridyl rings in the title compound, C_11_H_8_ClN_3_O_2_, is 22.65 (10)°, indicating a twisted mol­ecule. The amine H and nitro O atoms form a donor–acceptor pair for an intra­molecular N—H⋯O hydrogen bond so that the nitro group is almost coplanar with the pyridine ring to which it is connected [O—N—C—C torsion angle = 7.4 (3)°]. The pyridine N and Cl atoms are approximately *syn*. The crystal packing features C—H⋯Cl inter­actions that lead to undulating supra­molecular chains along [101]. These are connected into sheets by π–π inter­actions occurring between the benzene rings and between the pyridine rings of translationally related mol­ecules along the *b* axis [centroid–centroid distances = length of *b* axis = 3.7157 (2) Å].

## Related literature

For the structure of a related pyrimidine amine derivative, see: Aznan Akhmad *et al.* (2010 ▶).
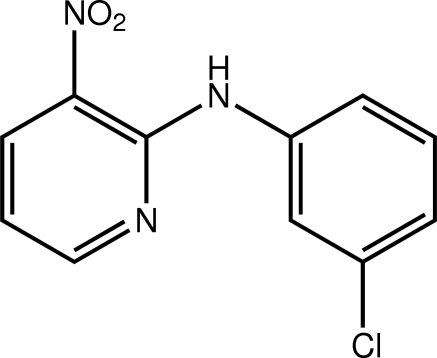


## Experimental

### 

#### Crystal data

C_11_H_8_ClN_3_O_2_
*M* *_r_* = 249.65
Monoclinic, 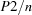

*a* = 15.8781 (10) Å
*b* = 3.7157 (2) Å
*c* = 18.0651 (13) Å
β = 102.252 (6)°
*V* = 1041.53 (11) Å^3^
*Z* = 4
Cu *K*α radiation
μ = 3.21 mm^−1^
*T* = 100 K
0.20 × 0.05 × 0.03 mm

#### Data collection

Agilent SuperNova Dual diffractometer with an Atlas detector
Absorption correction: multi-scan (*CrysAlis PRO*; Agilent, 2010 ▶) *T*_min_ = 0.566, *T*_max_ = 0.910
3341 measured reflections
1971 independent reflections
1684 reflections with *I* > 2σ(*I*)
*R*_int_ = 0.026

#### Refinement

*R*[*F* ^2^ > 2σ(*F* ^2^)] = 0.041
*wR*(*F* ^2^) = 0.119
*S* = 1.06
1971 reflections
158 parameters
H atoms treated by a mixture of independent and constrained refinement
Δρ_max_ = 0.25 e Å^−3^
Δρ_min_ = −0.34 e Å^−3^

### 

Data collection: *CrysAlis PRO* (Agilent, 2010 ▶); cell refinement: *CrysAlis PRO*; data reduction: *CrysAlis PRO*; program(s) used to solve structure: *SHELXS97* (Sheldrick, 2008 ▶); program(s) used to refine structure: *SHELXL97* (Sheldrick, 2008 ▶); molecular graphics: *ORTEP-3* (Farrugia, 1997 ▶) and *DIAMOND* (Brandenburg, 2006 ▶); software used to prepare material for publication: *publCIF* (Westrip, 2010 ▶).

## Supplementary Material

Crystal structure: contains datablock(s) global, I. DOI: 10.1107/S1600536811044011/hb6466sup1.cif

Structure factors: contains datablock(s) I. DOI: 10.1107/S1600536811044011/hb6466Isup2.hkl

Supplementary material file. DOI: 10.1107/S1600536811044011/hb6466Isup3.cml

Additional supplementary materials:  crystallographic information; 3D view; checkCIF report

## Figures and Tables

**Table 1:** Hydrogen-bond geometry (Å, °)

*D*—H⋯*A*	*D*—H	H⋯*A*	*D*⋯*A*	*D*—H⋯*A*
N1—H1*n*⋯O1	0.88 (3)	1.94 (3)	2.647 (2)	137 (2)
C9—H9⋯Cl1^i^	0.95	2.79	3.665 (2)	153

Symmetry code: (i) 
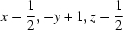
.

**Fig. 1.**
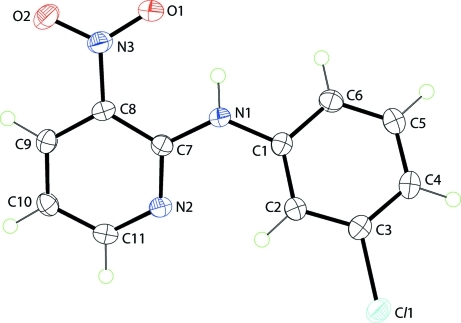
The molecular structure of (I) showing displacement ellipsoids at the 50% probability level.

**Fig. 2.**
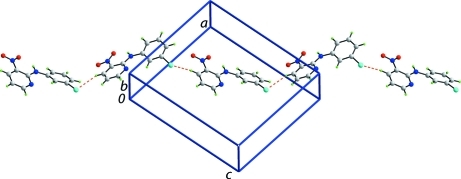
Undulating supramolecular chain along [101] in (I) sustained by C—H···Cl interactions, shown as orange dashed lines.

**Fig. 3.**
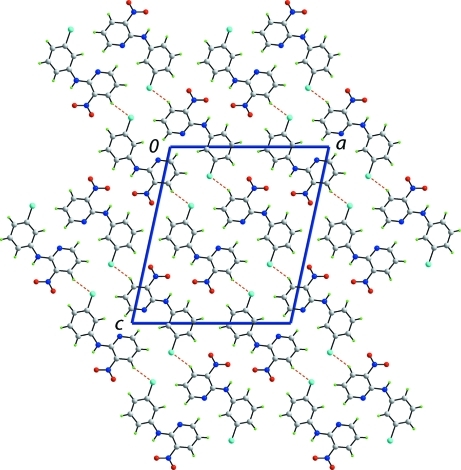
Unit-cell contents for (I) shown in projection down the b axis highlighting the stacking of chains. The C—H···Cl interactions are shown as orange dashed lines.

**Fig. 4.**
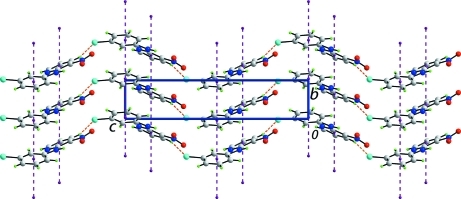
Stacking of chains highlighting the π–π interactions shown as purple dashed lines. The C—H···Cl interactions are shown as orange dashed lines.

## supplementary crystallographic information

### Comment

The title compound, (I), was investigated in connection with our ongoing crystallographic studies of pyrimidine derivatives (Aznan Akhmad *et al.*, 2010).

The molecule of (I), is twisted as seen in the value of the dihedral angle formed between the benzene and pyridyl rings of 22.65 (10)°. The nitro group is close to being co-planar with the pyridyl ring to which it is connected; the O1—N3—C8—C7 torsion angle is 7.4 (3)°. This conformation is stabilized by an intramolecular N1—H1*n*···O1 hydrogen bond, Table 1. The pyridine-N2 and *m*-Cl atoms are approximately *syn*.

In the crystal structure, C—H···Cl interactions, Table 1, lead to supramolecular chains with an undulating topology along [101], Fig. 2. These stack along the *b* axis, Fig. 3, whereby the components of the stacks are linked by π–π interactions occurring between translationally related benzene rings and between translationally related pyridyl rings with centroid···centroid distances corresponding to the *b* axis, *i.e*. = 3.7157 (2) Å, Fig. 4.

### Experimental

2-Chloro-3-nitro-pyridine (0.5 g, 0.00315 mol) and *m*-chloroaniline (0.3311 ml, 0.00315 mol) were refluxed in ethanol (5 ml) for 4 h at 385 K. The mixture was cooled and the obtained residue dissolved in a minimum volume of water (10 ml) and extracted with ether (3 *x* 10 ml). The ethereal layer was washed with water and dried over anhydrous sodium sulfate. Evaporation gave a reddish solid and recrystallization using diethyl ether yielded dark-orange prisms after one day. *M*.pt.: 406–409 K.

### Refinement

Carbon-bound H-atoms were placed in calculated positions (C—H 0.95 Å) and were included in the refinement in the riding model approximation, with *U*_iso_(H) set to 1.2*U*_equiv_(C). The N-bound H-atom was located in a difference Fourier map and its position and *U*_iso_ refined.

### Crystal data

**Table:**

C_11_H_8_ClN_3_O_2_	*F*(000) = 512
*M**_r_* = 249.65	*D*_x_ = 1.592 Mg m^−^^3^
Monoclinic, *P*2/*n*	Cu *K*α radiation, λ = 1.54184 Å
Hall symbol: -P 2yac	Cell parameters from 1441 reflections
*a* = 15.8781 (10) Å	θ = 2.9–74.2°
*b* = 3.7157 (2) Å	µ = 3.21 mm^−^^1^
*c* = 18.0651 (13) Å	*T* = 100 K
β = 102.252 (6)°	Prism, orange
*V* = 1041.53 (11) Å^3^	0.20 × 0.05 × 0.03 mm
*Z* = 4	

### Data collection

**Table:**

Agilent SuperNova Dual diffractometer with an Atlas detector	1971 independent reflections
Radiation source: SuperNova (Cu) X-ray Source	1684 reflections with *I* > 2σ(*I*)
Mirror	*R*_int_ = 0.026
Detector resolution: 10.4041 pixels mm^-1^	θ_max_ = 70.0°, θ_min_ = 3.4°
ω scan	*h* = −19→16
Absorption correction: multi-scan (*CrysAlis PRO*; Agilent, 2010)	*k* = −4→2
*T*_min_ = 0.566, *T*_max_ = 0.910	*l* = −22→20
3341 measured reflections	

### Refinement

**Table:**

Refinement on *F*^2^	Primary atom site location: structure-invariant direct methods
Least-squares matrix: full	Secondary atom site location: difference Fourier map
*R*[*F*^2^ > 2σ(*F*^2^)] = 0.041	Hydrogen site location: inferred from neighbouring sites
*wR*(*F*^2^) = 0.119	H atoms treated by a mixture of independent and constrained refinement
*S* = 1.06	*w* = 1/[σ^2^(*F*_o_^2^) + (0.0688*P*)^2^ + 0.3527*P*] where *P* = (*F*_o_^2^ + 2*F*_c_^2^)/3
1971 reflections	(Δ/σ)_max_ = 0.001
158 parameters	Δρ_max_ = 0.25 e Å^−^^3^
0 restraints	Δρ_min_ = −0.34 e Å^−^^3^

### Special details

**Table:**

Geometry. All s.u.'s (except the s.u. in the dihedral angle between two l.s. planes) are estimated using the full covariance matrix. The cell s.u.'s are taken into account individually in the estimation of s.u.'s in distances, angles and torsion angles; correlations between s.u.'s in cell parameters are only used when they are defined by crystal symmetry. An approximate (isotropic) treatment of cell s.u.'s is used for estimating s.u.'s involving l.s. planes.
Refinement. Refinement of *F*^2^ against ALL reflections. The weighted *R*-factor *wR* and goodness of fit *S* are based on *F*^2^, conventional *R*-factors *R* are based on *F*, with *F* set to zero for negative *F*^2^. The threshold expression of *F*^2^ > σ(*F*^2^) is used only for calculating *R*-factors(gt) *etc*. and is not relevant to the choice of reflections for refinement. *R*-factors based on *F*^2^ are statistically about twice as large as those based on *F*, and *R*- factors based on ALL data will be even larger.

### Fractional atomic coordinates and isotropic or equivalent isotropic displacement parameters (Å^2^)

**Table:**

	*x*	*y*	*z*	*U*_iso_*/*U*_eq_	
Cl1	0.78774 (3)	0.02226 (14)	0.66591 (3)	0.02816 (19)	
O1	0.66072 (10)	0.4237 (5)	0.23466 (9)	0.0357 (4)	
O2	0.54073 (10)	0.6787 (5)	0.18194 (9)	0.0348 (4)	
N1	0.68353 (11)	0.1981 (5)	0.37651 (10)	0.0227 (4)	
N2	0.55898 (11)	0.1975 (5)	0.42492 (9)	0.0221 (4)	
N3	0.58523 (12)	0.5144 (5)	0.23471 (10)	0.0259 (4)	
C1	0.74388 (13)	0.0748 (6)	0.44066 (12)	0.0217 (4)	
C2	0.73308 (13)	0.1013 (5)	0.51495 (12)	0.0214 (4)	
H2	0.6815	0.1947	0.5261	0.026*	
C3	0.80060 (14)	−0.0138 (5)	0.57217 (12)	0.0225 (5)	
C4	0.87682 (14)	−0.1519 (6)	0.55902 (13)	0.0258 (5)	
H4	0.9216	−0.2272	0.5997	0.031*	
C5	0.88592 (14)	−0.1769 (6)	0.48458 (12)	0.0268 (5)	
H5	0.9376	−0.2719	0.4738	0.032*	
C6	0.82038 (14)	−0.0648 (6)	0.42579 (13)	0.0245 (5)	
H6	0.8274	−0.0828	0.3750	0.029*	
C7	0.59821 (13)	0.2730 (5)	0.36741 (11)	0.0205 (4)	
C8	0.54874 (14)	0.4251 (6)	0.29929 (11)	0.0213 (4)	
C9	0.46195 (14)	0.4956 (5)	0.29291 (12)	0.0228 (5)	
H9	0.4289	0.5972	0.2477	0.027*	
C10	0.42391 (13)	0.4171 (6)	0.35261 (12)	0.0237 (5)	
H10	0.3646	0.4644	0.3500	0.028*	
C11	0.47531 (13)	0.2665 (6)	0.41660 (11)	0.0239 (5)	
H11	0.4489	0.2081	0.4576	0.029*	
H1n	0.7042 (17)	0.237 (8)	0.3359 (15)	0.041 (8)*	

### Atomic displacement parameters (Å^2^)

**Table:**

	*U*^11^	*U*^22^	*U*^33^	*U*^12^	*U*^13^	*U*^23^
Cl1	0.0287 (3)	0.0341 (3)	0.0202 (3)	−0.0026 (2)	0.0018 (2)	0.0004 (2)
O1	0.0280 (8)	0.0557 (11)	0.0255 (9)	0.0049 (8)	0.0110 (7)	0.0076 (8)
O2	0.0308 (8)	0.0474 (10)	0.0249 (9)	0.0005 (8)	0.0030 (7)	0.0135 (8)
N1	0.0213 (8)	0.0297 (9)	0.0180 (9)	0.0012 (7)	0.0060 (7)	0.0031 (8)
N2	0.0238 (9)	0.0234 (8)	0.0194 (9)	−0.0014 (7)	0.0054 (7)	−0.0001 (7)
N3	0.0267 (10)	0.0301 (10)	0.0214 (10)	−0.0029 (8)	0.0061 (8)	0.0015 (8)
C1	0.0215 (10)	0.0202 (9)	0.0225 (11)	−0.0019 (8)	0.0025 (8)	0.0005 (8)
C2	0.0207 (10)	0.0193 (9)	0.0242 (11)	−0.0010 (8)	0.0049 (8)	0.0001 (8)
C3	0.0249 (10)	0.0184 (10)	0.0229 (11)	−0.0052 (8)	0.0020 (8)	−0.0003 (8)
C4	0.0230 (10)	0.0223 (10)	0.0294 (12)	0.0000 (9)	−0.0007 (8)	0.0018 (9)
C5	0.0221 (10)	0.0254 (10)	0.0327 (12)	0.0026 (9)	0.0055 (9)	−0.0006 (9)
C6	0.0257 (11)	0.0232 (10)	0.0256 (11)	−0.0004 (9)	0.0078 (9)	−0.0021 (8)
C7	0.0215 (10)	0.0186 (9)	0.0217 (10)	−0.0008 (8)	0.0057 (8)	−0.0017 (8)
C8	0.0255 (10)	0.0211 (9)	0.0177 (10)	−0.0020 (8)	0.0056 (8)	0.0004 (8)
C9	0.0231 (10)	0.0211 (10)	0.0224 (11)	0.0002 (8)	0.0008 (8)	−0.0001 (8)
C10	0.0198 (10)	0.0239 (10)	0.0275 (11)	−0.0005 (8)	0.0055 (8)	−0.0036 (9)
C11	0.0259 (10)	0.0243 (11)	0.0234 (11)	−0.0028 (9)	0.0097 (8)	−0.0026 (9)

### Geometric parameters (Å, °)

**Table:**

Cl1—C3	1.753 (2)	C3—C4	1.381 (3)
O1—N3	1.245 (2)	C4—C5	1.386 (3)
O2—N3	1.222 (2)	C4—H4	0.9500
N1—C7	1.358 (3)	C5—C6	1.384 (3)
N1—C1	1.414 (3)	C5—H5	0.9500
N1—H1n	0.88 (3)	C6—H6	0.9500
N2—C11	1.330 (3)	C7—C8	1.428 (3)
N2—C7	1.349 (3)	C8—C9	1.383 (3)
N3—C8	1.447 (3)	C9—C10	1.374 (3)
C1—C2	1.392 (3)	C9—H9	0.9500
C1—C6	1.398 (3)	C10—C11	1.384 (3)
C2—C3	1.389 (3)	C10—H10	0.9500
C2—H2	0.9500	C11—H11	0.9500
			
C7—N1—C1	130.58 (17)	C6—C5—H5	119.7
C7—N1—H1n	113.9 (18)	C4—C5—H5	119.7
C1—N1—H1n	115.5 (18)	C5—C6—C1	120.4 (2)
C11—N2—C7	119.09 (18)	C5—C6—H6	119.8
O2—N3—O1	122.13 (18)	C1—C6—H6	119.8
O2—N3—C8	118.63 (18)	N2—C7—N1	118.39 (19)
O1—N3—C8	119.25 (18)	N2—C7—C8	119.10 (18)
C2—C1—C6	120.08 (19)	N1—C7—C8	122.50 (18)
C2—C1—N1	124.51 (19)	C9—C8—C7	120.17 (19)
C6—C1—N1	115.33 (18)	C9—C8—N3	116.86 (19)
C3—C2—C1	117.52 (19)	C7—C8—N3	122.97 (18)
C3—C2—H2	121.2	C10—C9—C8	119.4 (2)
C1—C2—H2	121.2	C10—C9—H9	120.3
C4—C3—C2	123.5 (2)	C8—C9—H9	120.3
C4—C3—Cl1	118.72 (17)	C9—C10—C11	117.50 (19)
C2—C3—Cl1	117.73 (16)	C9—C10—H10	121.3
C3—C4—C5	117.9 (2)	C11—C10—H10	121.3
C3—C4—H4	121.1	N2—C11—C10	124.77 (18)
C5—C4—H4	121.1	N2—C11—H11	117.6
C6—C5—C4	120.5 (2)	C10—C11—H11	117.6
			
C7—N1—C1—C2	−19.5 (4)	C1—N1—C7—C8	174.6 (2)
C7—N1—C1—C6	163.8 (2)	N2—C7—C8—C9	0.4 (3)
C6—C1—C2—C3	0.2 (3)	N1—C7—C8—C9	179.9 (2)
N1—C1—C2—C3	−176.37 (19)	N2—C7—C8—N3	−179.92 (19)
C1—C2—C3—C4	−0.1 (3)	N1—C7—C8—N3	−0.5 (3)
C1—C2—C3—Cl1	179.50 (15)	O2—N3—C8—C9	7.4 (3)
C2—C3—C4—C5	−0.2 (3)	O1—N3—C8—C9	−172.91 (19)
Cl1—C3—C4—C5	−179.74 (16)	O2—N3—C8—C7	−172.3 (2)
C3—C4—C5—C6	0.3 (3)	O1—N3—C8—C7	7.4 (3)
C4—C5—C6—C1	−0.1 (3)	C7—C8—C9—C10	−0.1 (3)
C2—C1—C6—C5	−0.1 (3)	N3—C8—C9—C10	−179.82 (18)
N1—C1—C6—C5	176.77 (19)	C8—C9—C10—C11	−0.6 (3)
C11—N2—C7—N1	−179.43 (18)	C7—N2—C11—C10	−0.8 (3)
C11—N2—C7—C8	0.0 (3)	C9—C10—C11—N2	1.1 (3)
C1—N1—C7—N2	−5.9 (3)		

### Hydrogen-bond geometry (Å, °)

**Table:**

*D*—H···*A*	*D*—H	H···*A*	*D*···*A*	*D*—H···*A*
N1—H1n···O1	0.88 (3)	1.94 (3)	2.647 (2)	137 (2)
C9—H9···Cl1^i^	0.95	2.79	3.665 (2)	153

Symmetry codes: (i) *x*−1/2, −*y*+1, *z*−1/2.
